# Will I speak louder if I see you struggling to understand? Speech modifications in response to non-verbal visual cues of listening effort

**DOI:** 10.3758/s13423-026-02942-3

**Published:** 2026-06-15

**Authors:** Elena Gessa, Chiara Valzolgher, Elena Giovanelli, Tommaso Rosi, Clément Desoche, Alessandro Farnè, Francesco Pavani

**Affiliations:** 1 Center for Mind/Brain Sciences –, CIMeC, University of Trento, Corso Bettini 31, 38068 Rovereto, Italy; 2 Level Up S.R.L., Trento, Italy; 3 Integrative Multisensory Perception Action and Cognition Team (Impact), Lyon Neuroscience Research Center (CRNL), INSERM U1028, CNRS UMR5292, University Claude Bernard Lyon 1, Bron, France

**Keywords:** Speech production, Listening effort, Face-to-face interaction, Non-verbal communication

## Abstract

During face-to-face interactions, overt non-verbal cues from conversational partners can reflect the quality of the conversation. Here, we investigated the impact of specific visual non-verbal cues (i.e., facial expressions of confusion, approaching movements towards the speaker), on the speakers’ acoustic–phonetic adaptations typically employed to enhance clarity (i.e., speech rate, voice intensity, and pitch). Participants read sentences to a listener seated in a separate room and visible through a head-mounted display. Crucially, no auditory feedback was provided. Unknown to participants, the listener was a confederate whose behavior was pre-recorded in silence using a 360° video camera. The confederate simulated three different levels of listening effort: easy, medium, and hard. After each trial, participants rated the estimated listening effort and comprehension of the confederate, as well as their own speaking effort. In Experiment 1 (*N* = 22), participants were told their voice was transmitted with varying background noise levels, providing a rationale for potential listener difficulties. In Experiment 2 (*N* = 12), no mention of background noise was made, to further isolate the effects of visual cues. In both experiments, participants adjusted their speech to the seen listener’s behavior. Experiment 1 showed adaptations across all measured parameters, and significant modulations persisted for most parameters in Experiment 2. These adaptations were already present in the earliest phase of exposure to the visual effort cues and remained stable across the mini-block, suggesting a rapid and highly sensitive monitoring mechanism. These findings indicate that visible non-verbal cues of listening effort can elicit acoustic–phonetic adaptations, even in the absence of acoustic feedback or explicit communicative signals, highlighting their role in regulating communication and maintaining speech clarity.

**Supplementary Information:**

The online version contains supplementary material available at 10.3758/s13423-026-02942-3.

## Introduction

In real-world social interactions, mutual comprehension is frequently challenged by environmental noise, listener characteristics, and familiarity with the content (Pichora-Fuller et al., 2016). The key to successful conversation is the ability of interlocutors to monitor the effectiveness of communication and make the relevant adjustments when needed (Lane & Tranel, 1971). Speech comprehension thus depends upon the speakers’ self-auditory monitoring and the acoustic–phonetic clarity of the speech signal they produce (Siegel & Pick, 1974).

The capacity for individuals to monitor their vocal output while speaking was first demonstrated by Etienne Lombard (1911), who qualitatively described an adjustment in vocal loudness in response to background noise (i.e., the Lombard effect). Subsequent studies showed that this regulation encompasses other vocal parameters, such as voice pitch (Elman, 1981), spectral shifts toward medium frequencies (Garber et al., 1981; Letowski et al., 1993), slower speech rate (Hanley & Steer, 1949), and greater articulatory movements (Garnier et al., 2010). This global reorganization of speech produced in noisy listening conditions is known as Lombard speech (for a review, see Garnier et al., 2010). Such psychophysiological studies proposed that the Lombard speech is an uncontrollable, reflexive behavior – a neural response to perturbations within the audio-phonation feedback loop (Bauer et al., 2006; Leydon et al., 2003; Nonaka et al., 1997). This hypothesis was supported by results indicating that similar vocal adaptations to noise also occur in preschool children (Amazi & Garber, 1982; Siegel et al., 1976) and animals (Cynx et al., 1998; Sinnott et al., 1975).

This perspective began to shift from a primarily bottom-up driven perspective when later psycholinguistic studies demonstrated a clear perceptual benefit when processing Lombard speech (Dreher & O’Neill, 1957; Pittman & Wiley, 2001; Summers et al., 1988). Based on the well-documented increase in speech comprehension, an alternative (though not mutually exclusive) hypothesis emerged: that speech adaptations to noise are also driven by communication goals (Junqua, 1993; Lane & Tranel, 1971). As noted by Lane and Tranel (1971, p. 692), “the speaker does not change his voice level to communicate better with himself, but rather with others.” The communication hypothesis was further supported by studies demonstrating that individuals adapt their speech production to the specific needs of the listener, even in the absence of acoustic interference. For example, speakers clarify their production when directing speech to infants (Fernald, 1992; Gleitman et al., 1984; Lindblom, et al., 1992; Snow et al., 1977), non-native listeners (Ferguson, 1975; Freed, 1981; Papoušek & Hwang, 1991; Scarborough et al., 2007; for a recent review, see Piazza et al., 2022), children with learning disabilities (Bradlow et al., 2003), or hearing-impaired individuals (Bergeson et al., 2006; Knoll et al., 2015; Picheny et al., 1985, 1986). Notably, this adaptation occurs even when acoustic distortions are merely anticipated rather than actively experienced. For example, Hazan and Baker (2011) showed that speakers adjust the acoustic–phonetic characteristics of their speech based on the expected listener’s degraded input, despite receiving undistorted auditory feedback themself.

In everyday conversations, listeners’ speech processing demands vary dynamically due to factors such as motivation, fatigue, or situational novelty (Pichora-Fuller et al., 2016). These demands can fluctuate even in the absence of changes in the auditory environment, or when explicit knowledge of the listener’s cognitive and hearing status is unavailable. In such scenarios, verbal feedback from the conversational partner could potentially signal glitches in understanding (Gass & Varonis, 1985; Hazan & Baker, 2011). However, such verbal feedback often remains unspoken due to social desirability, especially in contexts involving social stigma, such as hearing loss (Collins, 2022; Wallhagen, 2010). Given the possible limitations to explicit verbal feedback, it is critical to determine which other conversational signals speakers can employ to infer the quality of the conversation and the listener’s specific communicative need.

Addressing this gap, recent conversational models (Collins, 2022) suggest that during face-to-face interactions, individuals can assess conversational quality from reciprocal overt behavior. Visual cues such as eye contact, nodding, facial expressions, or physical orientation convey information about the listener’s attention and processing state. Conversational partners monitor these expressed behaviors to infer the listener’s comprehension, allowing speakers to strategically adapt their communicative behavior to support understanding. Within this framework, speakers may rely on non-verbal listener behavior as an indirect indicator of both listening engagement and listening effort. Listening engagement (Hermann & Johnsrude, 2020) refers to the listener’s motivation to sustain understanding, whereas listening effort (Johnsrude & Rodd, 2016; Peelle, 2018; Pichora-Fuller et al., 2016) reflects the subjective experience of difficulty during speech processing. This view aligns with the reconceptualization of verbal interaction as inherently multimodal, highlighting the role of visual cues – such as facial expressions – in communicating social intent and comprehension state (for a review, see Holler, 2025).

Empirical support for this perspective comes from recent studies showing that exclusively visual signals of comprehension difficulty can influence the speaker’s behaviors. For instance, Hömke et al. (2018) demonstrated that subtle variations in listener’s eye-blink duration affect speakers’ responses, with longer blinks eliciting shorter verbal answers. Similarly, Hömke et al. (2025) showed that listeners’ eyebrow furrows – a common visual signal of difficulties in understanding – prompt speakers to produce longer and more elaborated responses, consistent with conversational repair mechanisms. Together, these findings indicate that speakers attend to fine-grained visual cues of listener difficulty and adjust their communicative behavior accordingly. Importantly, however, the adaptations observed in these studies primarily concern discourse-level adjustments related to conversational repair, leaving open the question of whether visual cues alone are sufficient to trigger a reorganization of the acoustic speech signal itself.

In the present study, we tested the hypothesis that communicative adjustments can arise in response to visual non-verbal cues of listening effort, focusing on low-level acoustic–phonetic modifications that typically characterize Lombard speech (i.e., changes in vocal intensity, pitch, duration). More specifically, we examine coordinated adjustments elicited by visual cues of listening effort expressed by the listener during a face-to-face interaction, in the absence of acoustic distortion, explicit knowledge of the listener’s cognitive or hearing status, and verbal feedback. We therefore use the umbrella term *Lombard speech-like adaptations* to refer to these low-level acoustic adjustments, without implying that they arise from the same noise-driven mechanisms traditionally associated with Lombard speech.

To test this hypothesis under controlled conditions, we designed an experimental paradigm in which speakers interacted with a visually accessible listener whose non-verbal behavior was systematically manipulated. Participants read sentences to a listener seated in a separate room and visible only through a virtual reality head-mounted display. Unknown to participants, the listener was, in fact, a confederate whose behavior was pre-recorded in silence using a 360° video camera. The confederate simulated three different levels of listening effort: easy, medium, and hard (an example trial video is available at: https://osf.io/vu3c7/). We focused on a subset of visible non-verbal cues known to reflect listening effort: facial expressions of confusion and approaching body movements (Hadley et al., 2019; Krahmer & Swerts, 2005; Ricci Bitti et al., 2014). Confusion is typically conveyed through lip corner depression, chin movement, and brown raising (as described by Ricci Bitti et al., 2014), and can be detected by both adults and children (Krahmer & Swerts, 2005). Body posture also adjusts in response to listening difficulties, with reduced distance from the talker observed under higher noise conditions (Hadley et al., 2019). Crucially, we tested the effect of these visual cues in the absence of acoustic modifications of the environment, to separate the contribution of visual from auditory cues in speech adjustments. Likewise, we avoided any direct conversational feedback from the listener.

We predicted that speakers would react to non-verbal signals of greater listening effort by eliciting Lombard speech-like adaptations – including slower speech rate, more pauses, and modulation of intensity and pitch. In Experiment 1, we informed the speakers that the listener was hearing them under varying background noise levels (which they could not hear; as in Hazan & Baker, 2011) to justify the observed non-verbal cues. In Experiment 2, no explanation was provided, to further isolate the influence of visual cues on speech.

## Experiment 1

### Methods

#### Participants

Twenty-one young adults with typical hearing were recruited (age: *M* = 24.19, *SD* = 3.78, range = 19–32 years; 15 females), mostly among students at the University of Lyon. All participants were native French speakers with normal or corrected-to-normal vision, self-reported typical hearing, and no history of neurological and psychiatric diseases. To estimate the sample size, we referred to Hazan and Baker (2011), in which speakers changed the parameters of their vocalization based on the assumed intelligibility of the signal to the listener, as here. The estimated effect size was *f* = 0.41, as computed with G*Power (version 3.1.9.7) from the mean and standard deviations for mean word duration. The resulting sample size to achieve 90% power was 15 participants (two-tailed alpha = 0.05).

All participants signed an informed consent before the experimental session, which was conducted according to the criteria of the Declaration of Helsinki (1964, amended in 2013) and approved by the French Ethics Committee of the National Institute of Health and Medical Research (CEEI/IRB00003888).

#### Stimuli

**Fig. 1:**
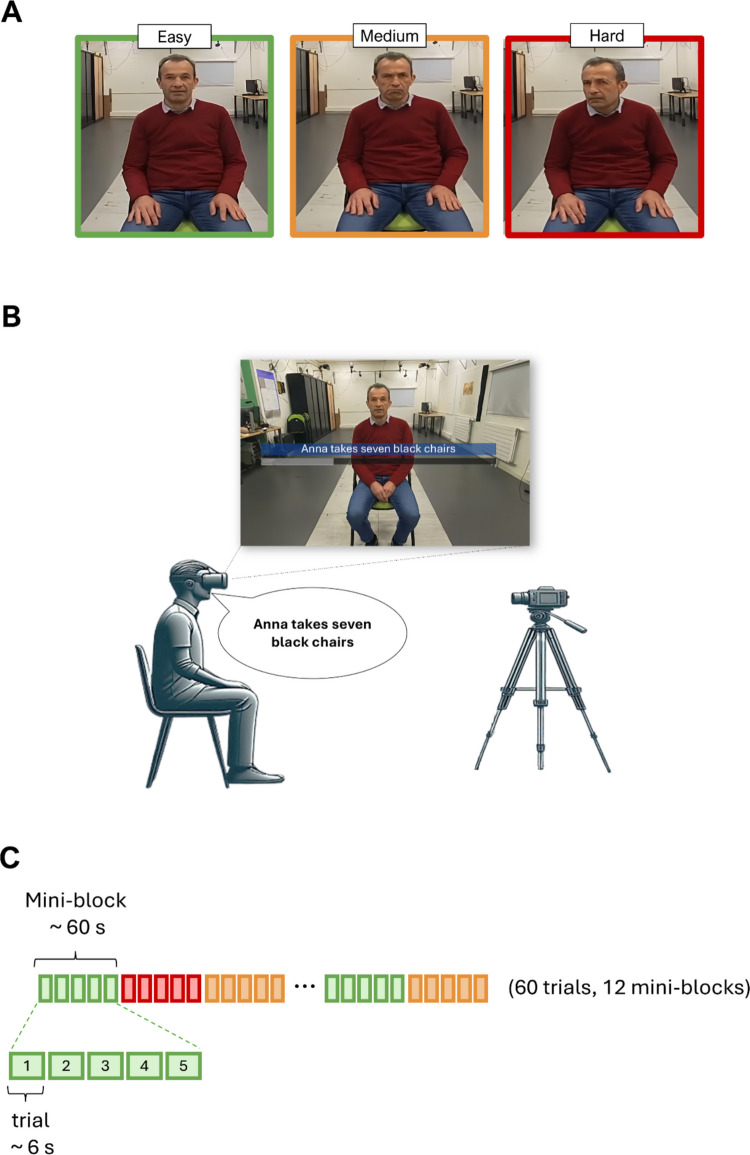
(**A**) Overt non-verbal cues of listening effort in each condition (green equals easy listening; yellow equals medium listening; and red equals hard listening); (**B**) graphic representation of the experimental setup with the participant seated on a chair, wearing the virtual reality (VR) helmet. The inset displays the visual scene visible during the trial (in this case an easy listening condition trial); (**C**) example of trial presentation, showing the mini-block structure of the experiment (order of mini-blocks changed across participants, see text for details)

Participants were exposed to 360-degree pre-recorded videos featuring a 60-year-old adult (from now on, the confederate) seated on a chair in an empty laboratory room. Videos were recorded using an Insta360 X3 camera (resolution: 8 K; sampling frequency: 48.0 kHz), placed at 1.50 m from the confederate to capture the full figure. Elevation of the camera was adjusted to be at the confederate eye-level to maximize participants’ face-to-face experience. Three conditions were designed by instructing the confederate to vary visible cues of listening effort: (1) easy listening, i.e., static posture, neutral expression; (2) medium listening, i.e., static posture, facial expressions of confusion; (3) hard listening, i.e., facial expressions of confusion and forward lean, reducing the physical distance from the camera (and approaching the participant in the video displayed in the head-mounted display, see Fig. 1A). Twenty videos were recorded per condition, for a total of 60 videos.

To provide the confederate with temporal pacing for displaying these pre-coded behaviors, five-word sentences were played through a loudspeaker positioned above the camera. In each trial, these sentences – identical to the phrases participants would later pronounce – were drawn from the French Matrix Sentence Test (Jansen et al., 2012). This clinical tool was used to minimize linguistic variability (i.e., syntax, word length, and word occurrence are balanced and fixed). Each video began and ended with the confederate facing the camera directly. To allow the confederate to return to the initial position at the end of each sentence, 1 s of silence preceded each sentence and 3 s followed it. Videos lasted an average of 6.64 s (± 0.19) to ensure participants had sufficient time to pronounce their speech during the experiment. After recording, the videos were cut to isolate the single trials and audio-deprived using DaVinci Resolve 17.

To assess whether the subset of the confederate visual non-verbal cues was associated with different levels of listening effort, we conducted an online survey involving 12 external raters. They evaluated the videos by estimating the confederate’s listening effort, speech comprehension, and level of attention (see Online Supplementary Material, Fig. 1). Separate repeated-measure ANOVAs were performed for each variable of interest, revealing a significant main effect of the listening condition in all cases (listening effort: *F*(2,22) = 34.91, *p* <.001, ${\eta }^{2}$ = 0.76; speech comprehension: *F*(1.16,12.79) = 14.16, *p* =.002, ${\eta }^{2}$ = 0.56; level of attention: *F*(1.34,14.70) = 4.53, *p* =.04, ${\eta }^{2}$ = 0.29). Crucially, the results confirmed the distinctiveness of each condition in terms of listening effort, as external raters perceived an increase in listening effort from easy to medium listening (*t*(11) = 3.86, ${p}_{FDR}$ <.001), from easy to hard listening (*t*(11) = 8.35, ${p}_{FDR}$ <.001), and from medium to hard listening (*t*(11) = 4.49, ${p}_{FDR}$ <.001). See Online Supplementary Materials for further details.

Videos were displayed in virtual reality (VR) on a 2.84-m diameter virtual sphere aligned with the VR helmet’s center, enhancing immersion and simulating shared physical space with the confederate. The 360-degree videos projected on the virtual sphere allowed for realistic visual adjustments in response to participants' body movements, simulating physical navigation within the space. This setup enabled participants to feel as if they could move around the environment and interact naturally with the confederate, thereby enhancing their engagement and realism in the simulated conversation.

#### Apparatus

The experiment was conducted using a Meta Quest 3 (128 GB; resolution: 2064 × 2208 per-eye; frequency: 72 Hz) linked to a computer (MSI, operating system Windows 10, Intel(R) Core (TM) i7-3770 CPU @ 4 GHz) via a link cable to display the VR environment. We recorded the participant’s speech directly from the built-in microphone (sampling rate: 48,000 Hz), positioned at the front side of the headset, ensuring proximity to the mouth.

During the experiment, participants selected their responses directly with their virtualized hands, without using the controllers. The virtual hands were visible in VR for the entire duration of the experimental session.

#### Procedure

Participants were informed that the experiment involved interacting with a listener located in an adjacent room and that they would need to read and then utter short sentences to the listener. They were told that their voice would be transmitted via a loudspeaker and that they would see the listener through the VR headset, while the listener viewed them via a video projected on a screen from the camera positioned in front of them (the Insta360 X3 camera also used for recordings of the stimuli). They were instructed to pronounce each sentence only once, without any other constraint. We informed them that the audio-visual connection between the two rooms would be active for a limited time (~ 6 s), and they needed to ensure that the target sentence was spoken within this time delay.

Participants were also told that background noise of varying intensity would play in the listener’s room, although they would not hear this noise during the experiment (as in Hazan & Baker, 2011). However, we did not specify how and when background noise levels would change over time. Their task was to speak as clearly as possible. See Fig. 1B for a graphic representation of the experimental setup. Original instructions in French are reported in the Open Science Framework (OSF) repository at: https://osf.io/vu3c7/overview?view_only=daf32cd52acb4731995132f0ceabfd16

Before the experiment, we collected measures of interpersonal closeness in consideration of prior work linking empathy to prosocial behavior (Eisenberg & Miller, 1987). Specifically, participants completed the Interpersonal Reactivity Index questionnaire (IRI, Gilet et al., 2013). In addition, they report their perceived closeness with the confederate through the Inclusion of Other in the Self scale (IOS, Aron et al., 1992), a single-item, pictorial index.

During the experiment, each trial began with a screen displaying the target sentence and a blurred image of the confederate (i.e., a screenshot of one of the videos). Participants were informed that the listener would neither see nor hear them between trials. Pressing the “Start” button in front of them triggered an audio-visual countdown of 3 s and initiated a 6-s video of the confederate. A loading bar was displayed at the foot of the confederate to provide participants with an estimation of the time remaining to pronounce the sentence. The target sentence to be spoken remained visible at the confederate’s chest level (see Fig. 1B). After each trial, the blurred image reappeared, signaling disconnection. Participants then rated the confederate’s listening effort, speech comprehension, and their own speaking effort. The measures related to the confederate's perceived listening effort and comprehension were collected to verify whether the effects of non-verbal visual cues observed in online studies with external raters would also emerge in a face-to-face communicative setting with our participants. This allowed us to confirm that our experimental manipulation of non-verbal behavior was effectively perceived by the participants as intended, thus validating the ecological relevance of our setup. Participants' self-reported speaking effort, on the other hand, was collected to assess their conscious awareness of adapting their speech.

To estimate the listening effort of the confederate, participants rated how much effort they thought the listener had exerted while processing the speech, using a Likert scale from 0 (no effort) to 10 (maximum effort). For speech comprehension, they estimated how much of the sentence the listener would likely be able to recall, using a Likert scale from 0 (no word correctly reported) to 10 (all words correctly reported). To evaluate speaking effort, participants rated their own effort in articulating the sentence on a Likert scale from 0 (no effort) to 10 (maximum effort).

Participants completed 60 trials (20 per condition), grouped in 12 mini-blocks of five consecutive trials from the same condition (Fig. 1C). The order of these mini-blocks was counterbalanced across participants (three orders), with each order starting and ending with a different condition. The aim of this choice was twofold: on the one hand, we reasoned that presenting the three conditions randomly would have prevented participants from predicting the listening effort in subsequent trials and adjusting their speech. On the other hand, we wanted to avoid a total separation between the three conditions. Since the confederate was pre-recorded and unresponsive, prolonged exposure to the same condition could discourage participants from adapting.

At the end of the experiment, participants were informed of the goals and the real design of the experiment. They were then asked to rate their belief in the cover story on a 7-point Likert scale (1 = not at all believable, 7 = completely believable). Each participant received a reimbursement of 10 euros for their participation.

#### Analyses

Audio registrations of each trial were pre-processed and analysed using MATLAB 2023b. First, we quantified the speed with which participants produced the sentences by calculating the speech rate, defined as the number of words (always five) per second of speech. We utilised the detectSpeech function to extract speech from each trial. Before processing all the trials, we visually examined sample plots of the selected speech segments to identify any error in our automatic procedure. Once we were confident that the function worked properly, we proceeded with the remaining trials. To better capture the actual time participants spoke, we also computed the proportion of speech within each trial. This required calculating the percentage of time occupied by speech within each 6-s trace (also known as phonation/time ratio; Towell et al., 1996). In addition, we computed the pause proportion, defined as the percentage of time corresponding to pauses between words, to examine whether articulatory patterns varied across conditions. 

Since the microphone was not calibrated, speech intensity could not be corrected for mouth-to-microphone distance. The Sound Pressure Level (SPL) of the speech was calculated using a frame-based approach with a window length of 125 ms (as in Bottalico et al., 2015), ensuring a fine-grained assessment of vocal intensity over time. For each frame, the root mean square (RMS) power was computed to obtain a measure of the average signal amplitude within the window. Next, the SPL for each frame was derived by converting the RMS power into decibels (dB) using a logarithmic transformation. Finally, to obtain a single SPL value representing the overall intensity of each trial, a logarithmic averaging of the frame-wise SPL values was performed. Finally, we calculated the mean fundamental frequency ($${f}_{0})$$ of the processed speech signal of each trial using the pitch function to detect increases in the vocal pitch as a function of non-verbal visual cues of listening effort. For each trial, average speech indexes (i.e., speech rate, speech proportion, pause proportion, voice intensity, and $${f}_{0}$$) were stored separately for each subject. The script for audio analyses can be retrieved from the OSF repository. 

The data were analyzed using linear mixed-effects (LME) models, executed in the statistical environment R via the lmer() function from the lme4 package. We fitted separate LME models for each of the acoustic–phonetic parameters (i.e., speech rate, speech proportion, pause proportion, voice intensity, and ${f}_{0}$) and the participant self-reported measures (i.e., speaking effort and perceived confederate’s listening effort and comprehension). In all models, listening condition was included as the primary predictor and modeled as a continuous numerical variable reflecting the graded amount of visible cues of listening effort displayed by the listener. This modeling choice was theoretically motivated by the structure of the experimental manipulation, in which visual information increased monotonically and cumulatively across conditions, from the absence of visual cues (easy listening), to facial expressions only (medium listening), to facial expressions combined with postural adjustments (hard listening). Treating the listening condition as a continuous predictor therefore captures the hypothesized graded relationship between the amount of visual information signaling listening effort and speakers’ adaptive responses. To assess the robustness of our findings, we re-ran all analyses treating listening condition as a categorical factor. This approach confirmed the main results, with all main effects of listening condition replicated across both experiments and showing the same pattern of effects. Differences were limited to a small number of marginal interactions in exploratory trial-level analyses, which did not affect the interpretation of the results (see Online Supplementary Materials for full details).

To account for the within-subjects design, the model included random intercepts for participants, as well as random slopes for the listening condition by participant (i.e., a maximal model structure of listening condition | participant). Since the exact same set of standardized sentences was presented across all listening conditions for every participant, item variability was controlled by using the same stimuli in all experimental conditions. 

Data visualization in R was performed with the ggplot2 package (Wickham et al., 2016).

## Results

**Fig. 2:**
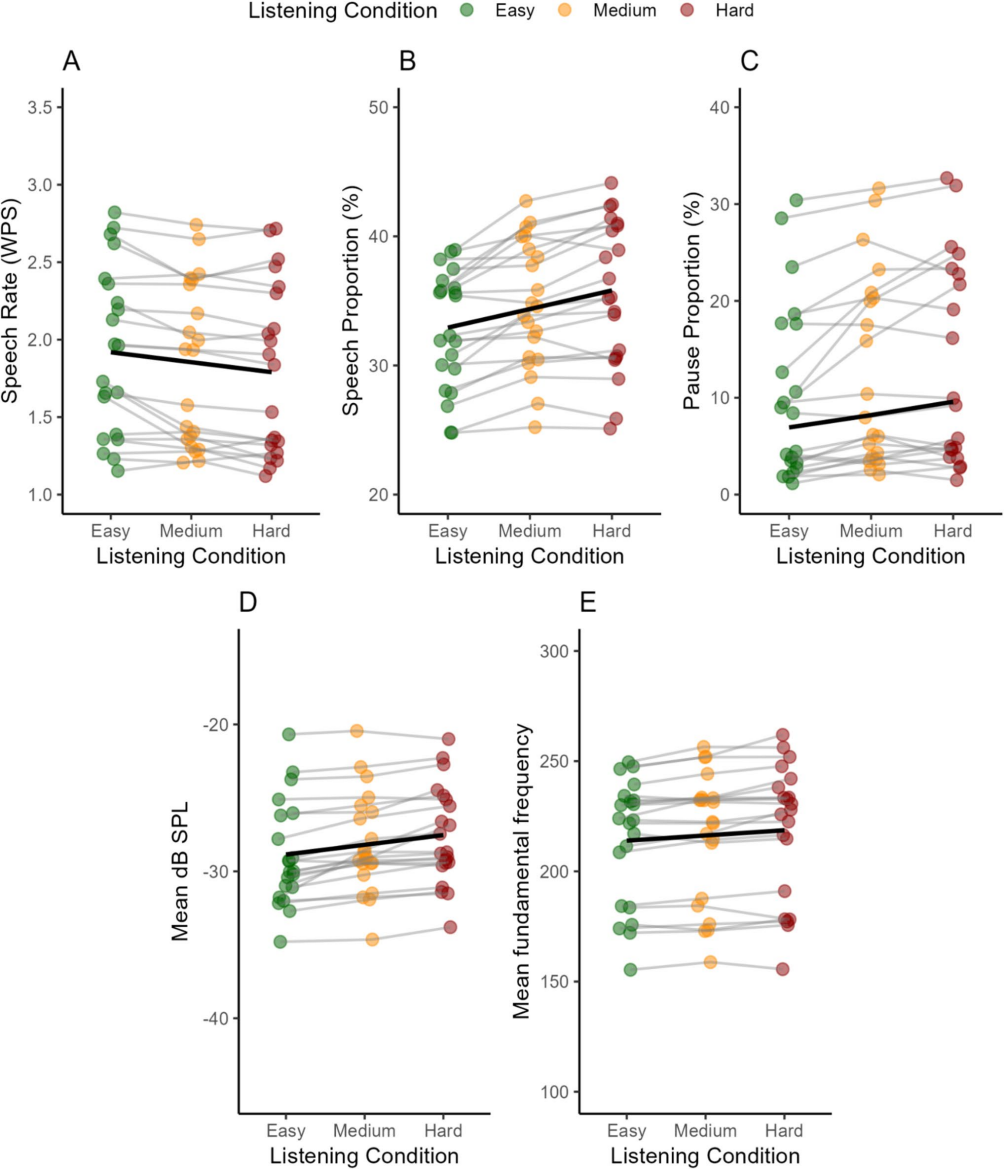
Changes in speech rate (**A**), speech proportion (**B**), pause proportion (**C**), mean voice intensity (**D**), and mean ${f}_{0}$ (**E**) as a function of the listening condition. The colored dots represent individual participant means for the dependent variable across listening conditions, with gray lines illustrating the within-subject trend. The black lines indicate the estimated mean trends for the Listening Condition

Figure 2 displays changes in speech rate, speech proportion, pause proportion, voice intensity, and ${f}_{0}$ as a function of listening condition. Speech rate significantly decreased with an increase in visual cues of listening effort (${X}^{2}$(1) = 16.48, *p* <.001, Fig. 2A). Similarly, speech proportion (${X}^{2}$(1) = 36.09, *p* <.001, Fig. 2B), pause proportion (${X}^{2}$(1) = 11.21, *p* <.001, Fig. 2C), voice intensity (${X}^{2}$(1) = 23.17, *p* <.001, Fig. 2D), and ${f}_{0}$ (${X}^{2}$(1) = 12.69, *p* <.001, Fig. 2E) significantly increased as visual cues of listening effort increased.

**Fig. 3:**
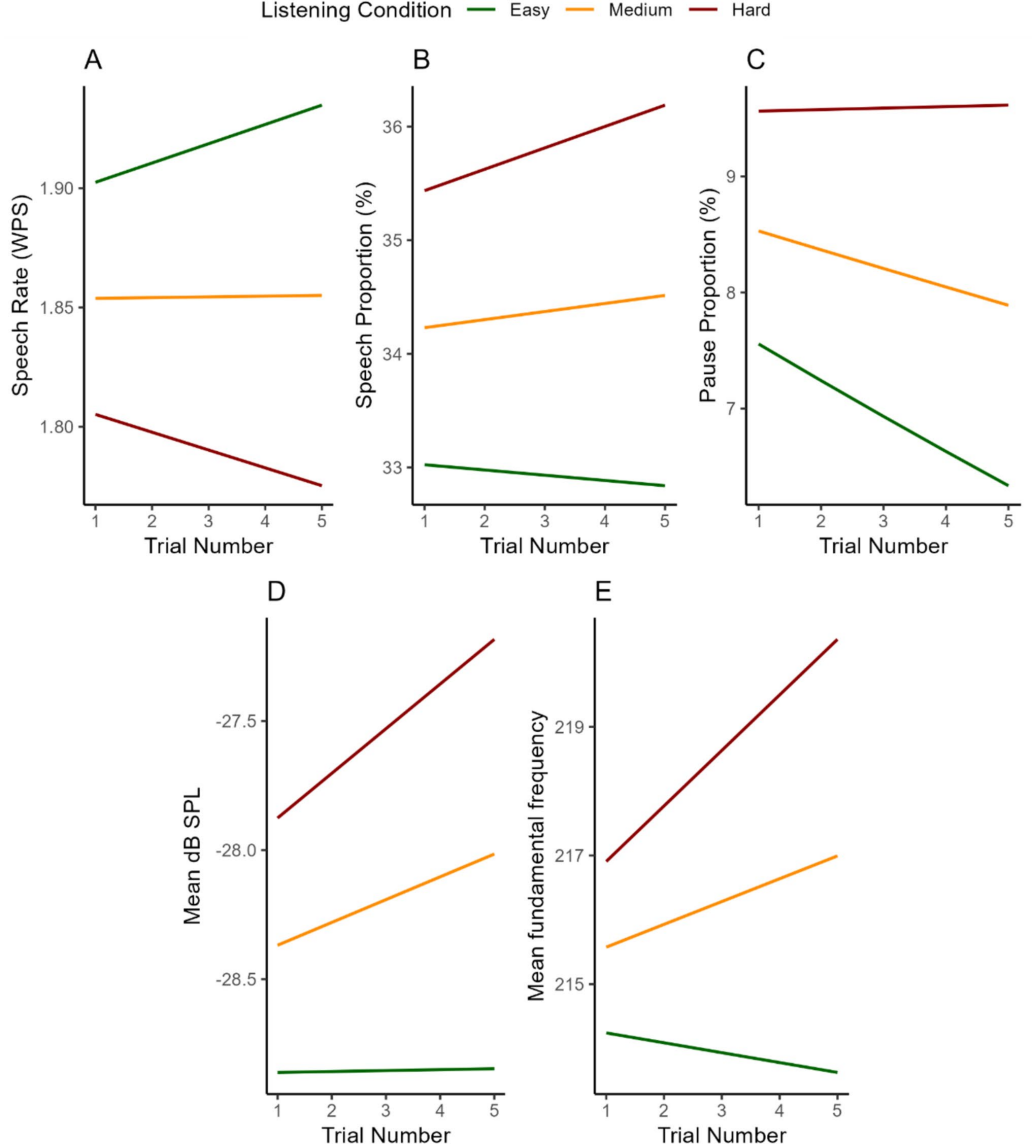
Changes in speech rate (**A**), speech proportion (**B**), pause proportion (**C**), mean voice intensity (**D**), and mean ${f}_{0}$ (**E**) as a function of trial number and listening condition. The colored lines indicate the estimated mean trends for the Listening Condition

To investigate the time course of Lombard speech-like adaptations and explore whether adaptation patterns changed during the experimental procedure, we examined how acoustic parameters changed as a function of trial position within each mini-block. Trial position, ranging from 1 to 5, indexed the sequential order of trials within a mini-block in which the same visual cues of listening effort were presented. This variable was entered as a continuous predictor in linear mixed-effects models together with listening condition and their interaction. A significant interaction between trial position and listening condition was interpreted as evidence that the trajectory of vocal adaptation across successive trials differed as a function of listening difficulty. Model-predicted values were subsequently inspected to characterize the direction and magnitude of these condition-specific changes (see Fig. 3).

For speech rate, neither a main effect of trial position (${X}^{2}$(1) = 1.26, *p* =.26), nor the interaction between trial position and listening condition was observed (${X}^{2}$(1) = 1.41, *p* =.23). A similar pattern of non-significance was observed for speech proportion (main effect:${X}^{2}$(1) = 0.29, *p* =.59; interaction: ${X}^{2}$(1) = 0.69, *p* =.41), and for pause proportion (main effect:${X}^{2}$(1) = 1.97, *p* =.16; interaction: ${X}^{2}$(1) = 1.07, *p* =.30). For ${f}_{0}$, no main effect of trial position was observed (${X}^{2}$(1) = 1.55, *p* =.21), but a significant interaction between trial position and listening condition emerged (${X}^{2}$(1) = 4.25, *p* =.04). Inspection of model-predicted values revealed that differences in ${f}_{0}$ across listening conditions became progressively more pronounced over successive trials, indicating a gradual adaptation of vocal pitch to listening conditions. Specifically, the difference in ${f}_{0}$ between the easy and hard listening conditions increased from approximately 2.7 Hz at trial 1 to 6.7 Hz at trial 5. Similarly, for voice intensity, no main effect of trial position was found (${X}^{2}$(1) = 1.13, *p* =.29), but the interaction with listening condition was significant (${X}^{2}$(1) = 5.77, *p* =.016). As with ${f}_{0},$ differences in vocal intensity across listening conditions became progressively more pronounced over successive trials, consistent with a gradual adaptation to listening demands. Specifically, the difference in intensity between the easy and hard listening conditions increased from approximately 1.0 dB at trial 1 to 1.7 dB at trial 5.

**Fig. 4:**
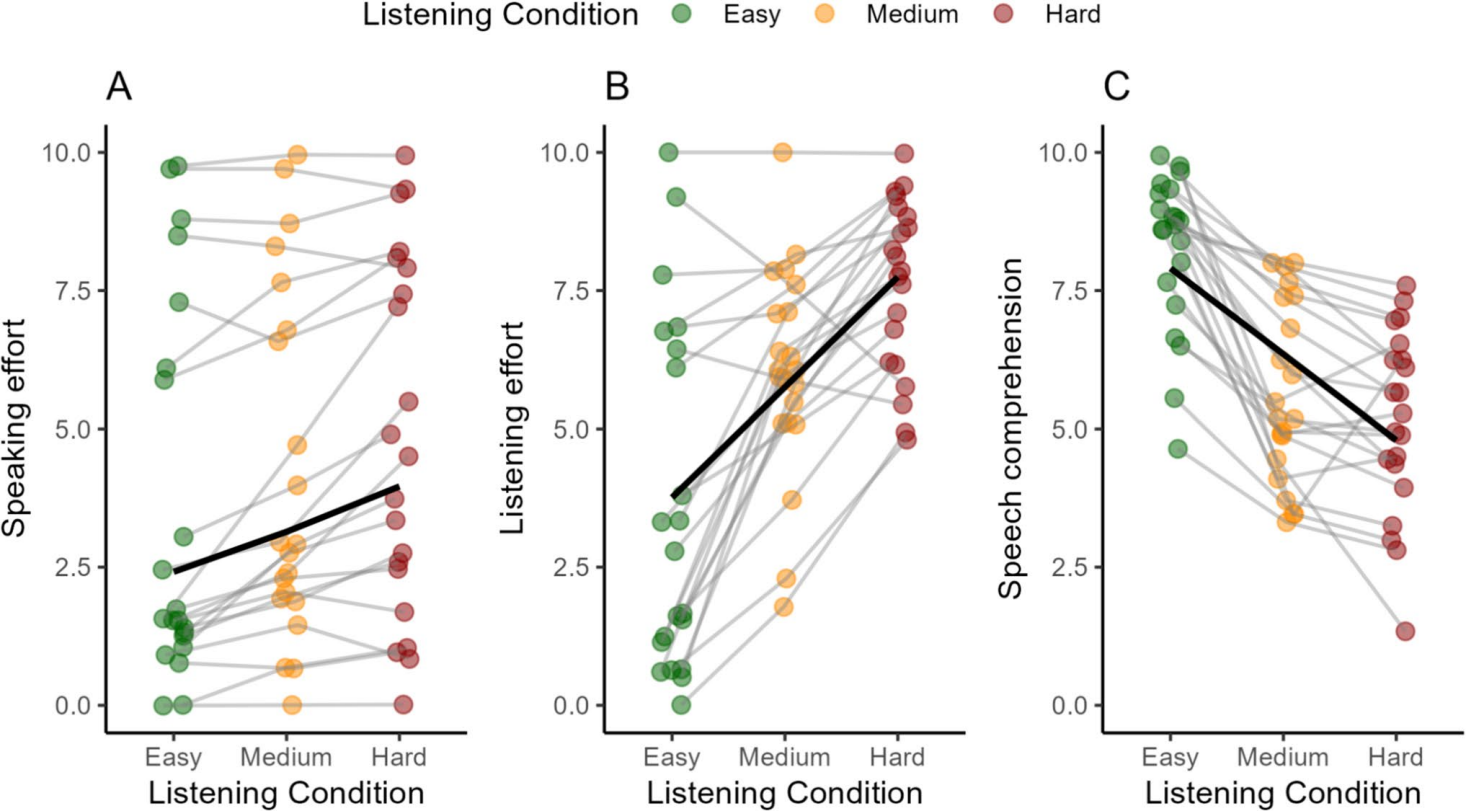
Speaking effort reported by participants as a function of the listening condition (**A**); estimated listening effort of the confederate as a function of the listening condition (**B**); estimated speech comprehension of the confederate as a function of the listening condition (**C**). The colored dots represent individual participant means for the dependent variable across listening conditions, with gray lines illustrating the within-subject trend. The black lines indicate the estimated mean trends for the Listening Condition

For what concerns the self-report measures, speech production changes were accompanied by increased speaking effort reported by participants as a function of the visible cues of listening effort (${X}^{2}$(1) = 17.05, *p* <.001; see Fig. 4A). In line with our manipulation, participants’ evaluations of the confederate’s listening effort also increased with visual cues of listening effort (${X}^{2}$(1) = 36.78, *p* <.001; Fig. 4B). Similarly, perceived speech comprehension of the confederate significantly decreased with an increase in visual cues of listening effort (${X}^{2}$(1) = 49.72, *p* <.001; Fig. 4C).

For completeness, we also explored whether perceived interpersonal closeness modulates the effect of the listener’s behavior on speech adaptations. To do so, we performed correlations between the IRI and the IOS measures (i.e., the Interpersonal Reactivity Index questionnaire and the Inclusion of the Other in the Self scale, respectively) and the difference between hard versus easy listening conditions measured for each of our dependent variables of interest. None of the correlations reached significance (all ${p}_{Bonf}$ >.49).

Finally, participants reported moderate belief in the cover story (*M* = 3.81, *SD* = 2.40). We analyzed the correlation between the reported belief in the cover story and the different acoustic–phonetic adjustments to visual cues of listening effort. None of the analyzed correlations were statistically significant (all ${ps}_{Bonf}$= 1.0, see Online Supplementary Material Fig. 2 for a full report).

## Discussion

Experiment 1 showed that participants adapted their speech in response to real-time visual cues of listening effort. Specifically, increasing visual cues of listening effort were associated with a significant increase in speech proportion, pause proportion, voice intensity and pitch, alongside a corresponding decrease in speech rate. For the majority of vocal parameters, Lombard speech-like adaptations to the visual cues were already present in the earliest phase of exposure to the visual effort cues and remained stable across the mini-block. The only exceptions were mean ${f}_{0}$ and vocal intensity, for which the observed changes across listening conditions became more pronounced across successive trials. Importantly, participants clearly distinguished between the increasing levels of listening effort of the confederate, and their self-reported speaking effort increased accordingly. The exploratory analyses on the link between Lombard speech-like adaptations and self-reported empathy or closeness did not yield any significant finding. Overall, the results of Experiment 1 show a global reorganization of speech, compatible with the speakers’ aim to maintain communicative clarity (Junqua, 1993; Lane & Tranel, 1971). Notably, these speech adaptations occurred despite the absence of acoustic feedback, prior knowledge of the listener’s cognitive and hearing status, and verbal feedback. Since all participants received identical instructions to “speak clearly” across conditions, the observed changes appear to result from their sensitivity to real-time visual non-verbal cues of listening effort produced by the listener.

One limitation to this conclusion is the fact that participants were informed of the presence of background noise in the adjacent room, to justify the visible confederate’s behavior. Although this implied no specific mention of noise levels, participants could have inferred escalating acoustic difficulties based on behavioral cues. Thus, speech adjustments might have resulted from a combination of sensitivity to visual signals and anticipated speech transmission degradation (i.e., a non-auditory cue, but related to knowledge of the listening context). Recall that Hazan and Baker (2011) found that speakers modulated their speech depending on the predicted (but not experienced) type of acoustic challenge faced by their interlocutor. To isolate further the effects of visual cues, Experiment 2 replicated the entire procedure without mentioning background noise in the listener’s experience. This removed explicit external explanations for the confederate’s behavior.

## Experiment 2

### Methods

#### Participants

Twelve young adults with typical hearing (age: *M* = 25.25, *SD* = 2.99, range = 22–33 years; five females) were recruited for Experiment 2, mostly among the students of the University of Lyon. They were all native French speakers, with normal or corrected-to-normal vision, reported typical hearing, and had no history of neurological and psychiatric diseases.

The sample size was determined based on the effect size observed in Experiment 1 specifically for voice intensity (*f* = 1.03). A G*Power analysis indicated that a minimum of six participants would be sufficient to detect the effect with 99% power. In Experiment 2, we conservatively doubled this sample size (*N* = 12) to ensure robust detection of the effect. None took part in Experiment 1.

#### Stimuli, apparatus, procedure, and analyses

These were identical to Experiment 1, except that participants were not informed about the confederate’s experience in the adjacent room, and no explanation was given for the listener’s varying behavior. Original instructions in French are reported in the OSF repository.

## Results

**Fig. 5:**
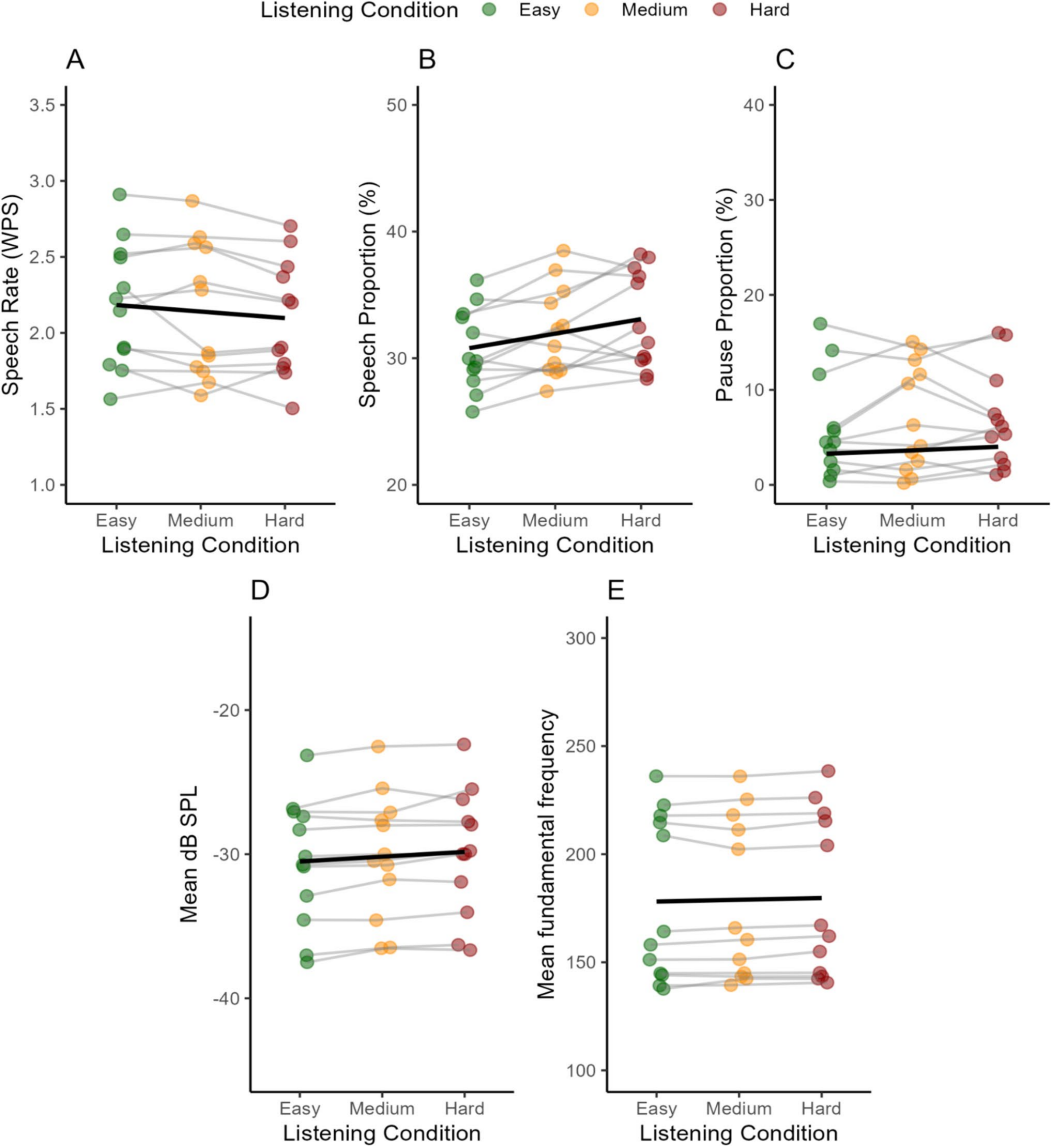
Changes in speech rate (**A**), speech proportion (**B**), pause proportion (**C**), mean voice intensity (**D**), and mean ${f}_{0}$ (**E**) as a function of the listening condition. The colored dots represent individual participant means for the dependent variable across listening conditions, with gray lines illustrating the within-subject trend. The black lines indicate the estimated mean trends for the Listening Condition

Figure 5 illustrates changes in speech rate, speech proportion, pause proportion, voice intensity, and ${f}_{0}$ as a function of the listening condition. Consistent with the findings of Experiment 1, speech rate significantly decreased with an increase in visual cues of listening effort (${X}^{2}$(1) = 6.07, *p* =.01; Fig. 5A). A similar pattern of increase with listening effort was found for speech proportion (${X}^{2}$(1) = 14.27, *p* <.001; Fig. 5B) and voice intensity (${X}^{2}$(1) = 19.71, *p* <.001; Fig. 5D). Conversely, the listening condition had no significant effect on pause proportion (${X}^{2}$(1) = 2.47, *p* =.12; Fig. 4C) or ${f}_{0}$ (${X}^{2}$(1) = 3.25, *p* =.07; Fig. 5E).

**Fig. 6:**
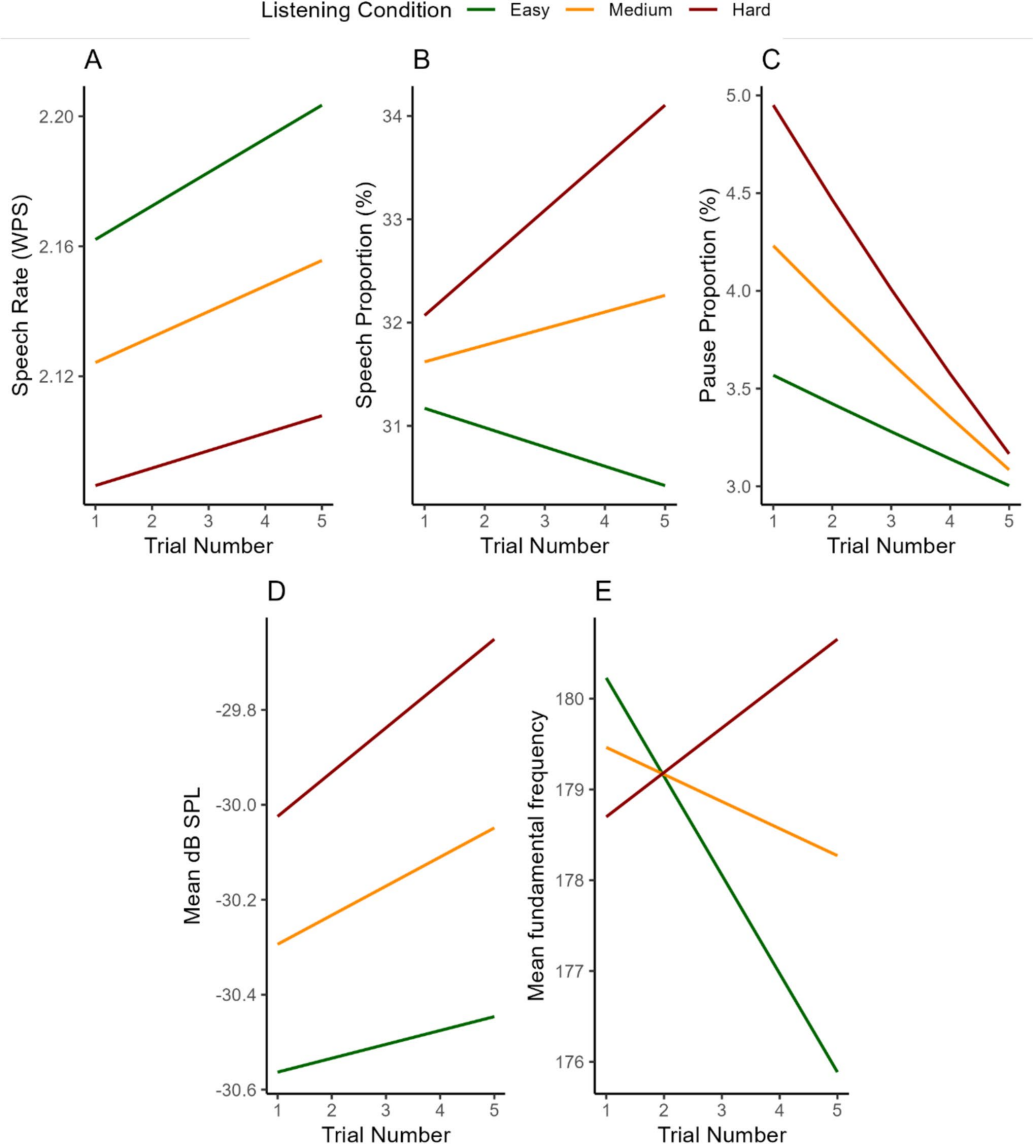
Changes in speech rate (**A**), speech proportion (**B**), pause proportion (**C**), mean voice intensity (**D**), and mean ${f}_{0}$ (**E**) as a function of trial number and listening condition. The colored lines indicate the estimated mean trends for the Listening Condition

As for Experiment 1, we also examined if adaptation patterns changed during the mini-block, incorporating trial position into the LME models’ structure (see Fig. 6). For the majority of the acoustic parameters examined, no significant main effect of trial position and no significant two-way interaction between trial position and the listening condition was observed. Specifically, this finding was registered for speech rate (main effect: ${X}^{2}$(1) = 0.31, *p* =.58; interaction: ${X}^{2}$(1) = 0.05, *p* =.82), pause proportion (main effect: ${X}^{2}$(1) = 0.0009, *p* =.98; interaction: ${X}^{2}$(1) = 0.73, *p* =.39), and voice intensity (main effect: ${X}^{2}$(1) = 0.0006, *p* =.98; interaction: ${X}^{2}$(1) = 0.37, *p* =.54). Conversely, a significant main effect of trial position was found for ${f}_{0}$ (${X}^{2}$(1) = 7.67, *p* =.006), indicating a small but consistent change across trials within the mini-block. Inspection of model-predicted values revealed a slight decrease in ${f}_{0}$ from trial 1 (≈179.5 Hz) to trial 5 (≈178.3 Hz). Moreover, a significant trial position x listening condition interaction emerged for ${f}_{0}$ (${X}^{2}$(1) = 6.33, *p* =.01). The differences in ${f}_{0}$ across listening conditions became progressively more pronounced over successive trials, consistent with a gradual adaptation to listening demands. Specifically, the difference in ${f}_{0}$ between the easy and hard listening conditions increased from approximately 1.53 Hz at trial 1 to 4.77 Hz at trial 5. Similarly, for speech proportion, the trial position x listening condition interaction was significant (${X}^{2}$(1) = 4.62, *p* =.03). Model-predicted values indicated that differences between easy and hard listening conditions became progressively more pronounced over successive trials: speech proportion differences increased from approximately 0.9% at trial 1 to 3.68% at trial 5. No main effect of mini-block part was observed for speech proportion (${X}^{2}$(1) = 2.34, *p* =.13).

**Fig. 7:**
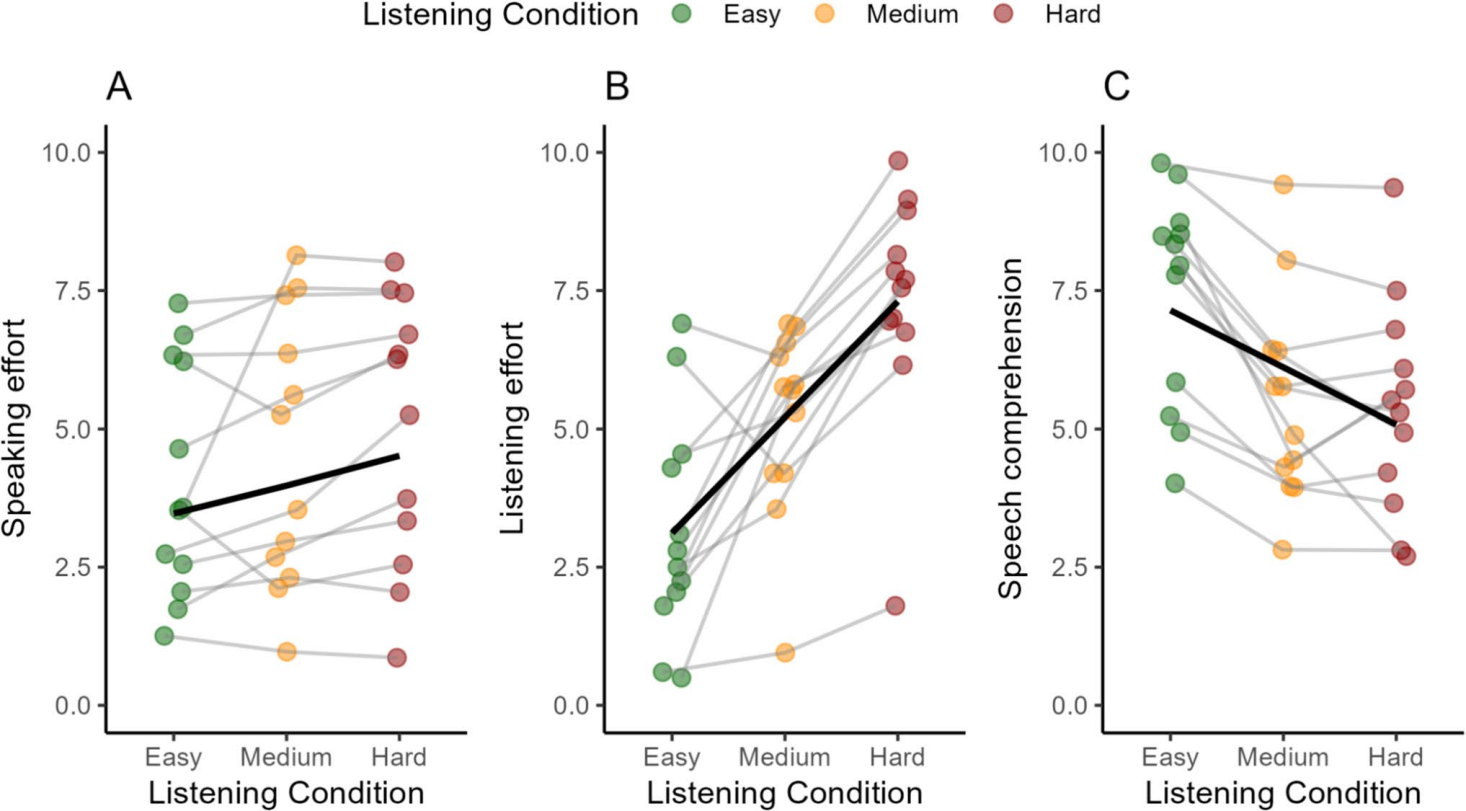
Speaking effort reported by participants as a function of the listening condition (**A**); estimated listening effort of the confederate as a function of the listening condition (**B**); estimated speech comprehension of the confederate as a function of the listening condition (**C**). The colored dots represent individual participant means for the dependent variable across listening conditions, with gray lines illustrating the within-subject trend. The black lines indicate the estimated mean trends for the Listening Condition

For self-report measures, speech production changes were again accompanied by increased speaking effort reported by participants as a function of the visual cues of listening effort (${X}^{2}$(1) = 4.42, *p* =.04; see Fig. 7A). Coherently, participants’ ratings of the confederate’s listening effort also increased with visual cues of listening effort (${X}^{2}$(1) = 35.31, *p* <.001; see Fig. 7B). Similarly, perceived speech comprehension of the confederate significantly decreased with an increase in visual cues of listening effort (${X}^{2}$(1) = 19.41, *p* <.001; see Fig. 7C).

Finally, as for Experiment 1, no significant correlation was observed between perceived interpersonal closeness and speech adaptations (all ${p}_{Bonf}$ >.30).

Participants reported low belief in the cover story (*M* = 1.91, *SD* = 1.93). However, the reported belief in the cover story did not correlate with the acoustic–phonetic adaptations of the participants (all ${ps}_{Bonf}$= 1.0, see Online Supplementary Material Fig. 3 for a full report).

## Discussion

Experiment 2 confirmed that Lombard speech-like adaptations emerge robustly in response to a listener’s visual cues of listening effort. Participants spontaneously modified their vocal production across multiple dimensions, specifically adjusting their speech rate, speech proportion, and speech intensity. While this suggests a global reorganization of the speech to enhance speech clarity (Junqua, 1993; Lane & Tranel, 1971), pause proportion and ${f}_{0}$ remained largely unaffected, indicating that the response to visual cues was limited to specific parameters of the vocal output. The temporal analysis further elucidated the dynamics of this adaptation, revealing that, for most parameters, the vocal adjustments were already present in the earliest phase of exposure to the visual effort cues and remained stable across the mini-block. However, participants displayed stronger adaptations to the non-verbal cues in the second half of the mini-block for ${f}_{0}$ and speech proportion adjustments, suggesting a time-dependent adaptation for these specific parameters.

Crucially, these Lombard speech-like adjustments were initiated solely by the visual cues of listening effort, emerging in the absence of acoustic feedback, prior knowledge of the listener’s status, or verbal interaction. This underscores the notion that visual signals alone are sufficient to drive adjustments in communication strategy. It is worth noting that Experiment 2 was conceived as a targeted follow-up study to test the robustness of the effects observed in Experiment 1 under more restrictive conditions. Despite the smaller sample, the patterns of adaptation observed in Experiment 2 were consistent with those n Experiment 1, both in magnitude and in temporal dynamics.

The general reorganization of the speech was mirrored in the self-reported speaking effort, which scaled with visual cues of listening effort. Consistent with Experiment 1, participants clearly distinguished between the increasing levels of listening effort of the confederate, which was reflected in their coherent ratings of perceived listening effort and speech comprehension of the listener. Finally, no systematic association was observed between speech modifications and participants’ empathy or perceived closeness.

## General discussion

Human communication critically depends on speakers’ ability to monitor the effectiveness of their own productions and to adapt them to the perceived needs of their interlocutor. While such monitoring has traditionally been studied through auditory feedback and explicit conversational signals, the present study demonstrates that speakers can also rely on subtle visual non-verbal cues to guide rapid and systematic acoustic–phonetic adaptations. These cues allow speakers to calibrate their speech in real time, even in the absence of acoustic interference or explicit instruction about the listener’s hearing state.

Listening, although central to communication, is here considered primarily as an inferred process: speakers adjust their production based on their perception of the listener’s effort rather than on direct measurement of listener comprehension. Building on this perspective, the present research investigated whether visual cues alone are sufficient to trigger complex acoustic–phonetic Lombard speech-like adaptations. While Lombard speech has traditionally been associated with auditory challenges such as background noise or reverberation (Garnier et al., 2010), the present findings show that visual signals of listening effort are sufficient to induce multi-parameter adjustments in speech rate, intensity, and pausing. Importantly, in the absence of acoustic interference, auditory feedback, explicit knowledge about the listener, or verbal interaction, no or minimal speech adaptation would have been a plausible outcome within feedback-based models of speech adaptation.

Our findings reveal that speakers adapt their speech flexibly depending on the context and perceived communicative need. In Experiment 1, a global reorganization of the acoustic signal occurred, including adjustments in speech rate, speech and pause proportion, intensity, and mean ${f}_{0}$. In Experiment 2, however, adaptation was less global: mean ${f}_{0}$ and pause proportion remained largely unchanged, while other parameters continued to reflect sensitivity to visual cues. This difference may suggest that speakers selectively modulate specific acoustic–phonetic dimensions depending on the perceived source and severity of the listener’s communicative challenge, with some features – such as speech rate and vocal intensity – remaining stable in the absence of explicit cues for communicative difficulty. Although a direct comparison of the two experiments was not the primary goal of our study, we report a combined analysis in the Online Supplementary Materials. The results revealed that the experimental condition (i.e., instruction type) did not lead to significant differences in the magnitude of the adjustments made as a function of the visual cues (all *p*s >.09). The only significant difference was a lower overall ${f}_{0}$ in Experiment 2, likely attributable to the higher proportion of male participants (Titze, 1989). Overall, the combined evidence from both experiments supports the conclusion that visual cues of listening effort are sufficient to guide systematic speech adjustments. Future research is needed to clarify how specific conditions modulate different acoustic–phonetic dimensions.

Crucially, adaptation occurred rapidly and scaled proportionally with the listener’s visible effort, suggesting the involvement of a sensitive monitoring response to visual social cues. While the observed changes were subtle and potentially non-perceptible to the human ear in a real-world setting, their graded nature indicates that they may reflect genuine, bottom-up responses to visual cues rather than purely top-down strategic adjustments driven by the artificial context or demand characteristics. While the communicative efficiency of these modulations may be limited, the fact that they were rapidly triggered and scaled proportionally to the perceived visual level of the listener’s effort underscores the sensitivity of the speaker’s monitoring mechanism for visual social feedback, even under artificial experimental constraints.

Compared to explicitly instructed clear speech, which is typically characterized by top-down, volitional effort to maximize intelligibility (e.g., marked reductions in speech rate and exaggerated articulation; Smiljanic & Bradlow, 2007), the adaptations observed here appear qualitatively different. First, the magnitude of these adjustments is relatively small and does not reflect the kind of exaggerated enhancement of clarity that characterizes clear speech. Second, rather than being implemented as a general communicative strategy, these modifications scale dynamically with the listener’s visible effort, suggesting a fine-grained, context-sensitive calibration of speech. Accordingly, the observed adaptations seem to reflect listener-driven adjustments rather than uniform, speaker-driven attempts to increase intelligibility.

Indeed, our findings share important similarities with listener-directed speech registers, such as hearing-impaired-, infant-, or foreigner-directed speech, where speakers adjust prosodic and temporal features to facilitate comprehension (Lane & Tranel, 1971). However, the present results point to a different source of control: whereas such adjustments are typically implemented based on generalized assumptions about the listener’s needs, the Lombard-speech-like adaptations observed here are modulated online by non-verbal visual cues of listening effort. Thus, they reflect a feedback-driven, dynamically updated calibration rather than an a priori communicative strategy.

Within this framework, visual signals of listening effort can be interpreted as functional indicators of communicative success (Collins, 2022), supporting a form of continuous, fine-grained calibration of speech production. Rather than reflecting a stable shift in speaking style, the observed modifications appear to constitute a dynamic, moment-to-moment calibration process. More broadly, our study integrates and extends recent conversational models (Collins, 2022; Holler & Levinson, 2019; Vigliocco et al., 2014), describing communication as an inherently multimodal process. In line with these frameworks, our findings suggest that speakers treat visual cues not merely as social signals, but as functional indicators of communicative success that guide online speech production. This perspective aligns with the idea of progressive interpersonal entrainment, where speakers dynamically adjust to the listener’s perceived effort to optimize communication (Fusaroli et al., 2014).

Limitations of the present study include the use of a single confederate of a fixed age, which may have influenced assumptions about hearing ability, and the absence of real-time social contingency due to pre-recorded video sequences. Future research could explore how the apparent age of the listener or the integration of live interactive avatars affects speech adaptation. Additionally, investigating co-speech gestures under visual listening cues could reveal further dimensions of multimodal adaptation, as gesture production is known to increase under communicative challenges (Ozyurek, 2002; Kim et al., 2024).

In conclusion, our results suggest speakers actively monitor the listener’s state and adapt their speech accordingly. Visual cues of listening effort alone can elicit rapid, graded, and systematic adjustments in acoustic–phonetic features, highlighting the speaker as an active agent in shaping communication. Listening, therefore, emerges as an inferred target of adaptation, underscoring the dynamic and multimodal nature of human interaction.

## Supplementary Information

Below is the link to the electronic supplementary material.

Supplementary file1 (DOCX 4529 kb)

## Data Availability

The data and materials for all experiments are available at https://osf.io/vu3c7/ and none of the experiments was preregistered.

## Code availability

The custom code for speech processing is available at https://osf.io/vu3c7/.
