# Supplementary material for: Will I speak louder if I see you struggling to understand? Speech modifications in response to non-verbal visual cues of listening effort
Source: Psychon Bull Rev. 2026 Jun 15;33(5):176. doi: 10.3758/s13423-026-02942-3 (PMC13269471; doi:10.3758/s13423-026-02942-3)
Supplement: Supplementary file 1 — Supplementary file1 (DOCX 4529 kb) [file 13423_2026_2942_MOESM1_ESM.docx]

**Supplementary Materials**

**Evaluation of the videos performed by the external raters**

To confirm the distinctiveness of each listening condition (i.e., easy, medium, and hard listening) based on the non-verbal cues of listening effort displayed by the confederate, we conducted an online survey on Qualtrics (version: April 2024) involving 12 external raters (age: M = 22.83, SD = 2.89, range [19-28], all females). All external raters were Italian young adults with typical hearing, had normal or corrected-to-normal vision and reported no history of neurological and psychiatric diseases.

The external raters were informed that they would have to carefully watch videos of an older adult performing a listening in noise task, but that they would not have access to the audio. The sixty videos were presented in a random order and the raters could view each video only once. Based on their observations, the raters were asked to estimate the older adult’s listening effort using a ruler scale from 0 (minimum level) to 100 (maximum level). Acknowledging that interpretations of listening effort may vary among individuals (Pichora-Fuller et al., 2016), we examined changes in the perceived attentional level and speech comprehension of the listener as depicted in the videos (as reported using two additional ruler scales from 0 to 100).

We performed a repeated measure ANOVA with listening condition (easy, medium, and hard listening) as a within-participant factor on the estimated listening effort. The analysis revealed a main effect of listening condition (*F*(2,22) = 34.91, *p* < .001, $\eta^{2}$ = .76; Supplementary Figure 1A). There was a significant reduction in the estimated listening effort from hard to medium (*t*(11) = 4.49, $p_{FDR}$ < .001), from medium to easy listening (*t*(11) = 3.86, $p_{FDR}$ < .001), as well as from hard to easy (*t*(11) = 8.35, $p_{FDR}$ < .001).

We performed the same repeated measure ANOVA on the perceived level of attention, which required Greenhouse-Geisser correction due to a violation of sphericity (as indicated by Maucly’s test). The analysis revealed a main effect of listening condition (*F*(1.34,14.70) = 4.53, *p* = .04, $\eta^{2}$ = .29; Supplementary Figure 1B). A significant increase in the estimated level of attention emerged only from easy to hard listening (*t*(11) = 2.97, $p_{FDR}$ = .02; easy vs medium: *t*(11) = 1.91, $p_{FDR}$ = .10; medium vs hard: *t*(11) = 1.06, $p_{FDR}$ = .30).

We performed a repeated measure ANOVA on the perceived speech comprehension, which required Greenhouse-Geisser correction due to a violation of sphericity (as indicated by Maucly’s test). The analysis revealed a main effect of listening condition (*F*(1.16,12.79) = 14.16, *p* = .002, $\eta^{2}$ = .56; Supplementary Figure 1C). A significant increase in the estimated speech comprehension emerged from easy to medium (*t*(11) = 5.03, $p_{FDR}$ < .001) and from easy to hard listening (*t*(11) = 3.95, $p_{FDR}$ = .001), while no difference emerged between hard and medium listening (*t*(11) = 1.11, $p_{FDR}$ = .28).


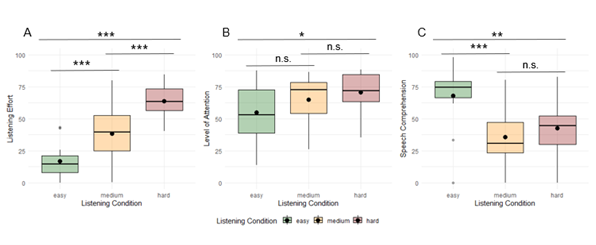


Supplementary Figure 1. A) Mean estimated listening effort of the confederate as a function of the listening condition (green equals easy listening; yellow equals medium listening; and red equals hard listening); B) mean estimated level of attention of the confederate as a function of the listening condition; C) mean estimated speech comprehension of the confederate as a function of the listening condition.

Additionally, within each video, the average level of listening effort reported across participants significantly positively correlated with their estimated level of attention for that video (*r* = 0.79, *p* < .001). Similarly, the average level of listening effort for each video significantly negatively correlated with the estimated speech comprehension of the confederate in the video (*r* = - 0.63, *p* < .001).

**Impact of Cover Story Credibility on Speech Adaptations: Experiment 1**

**
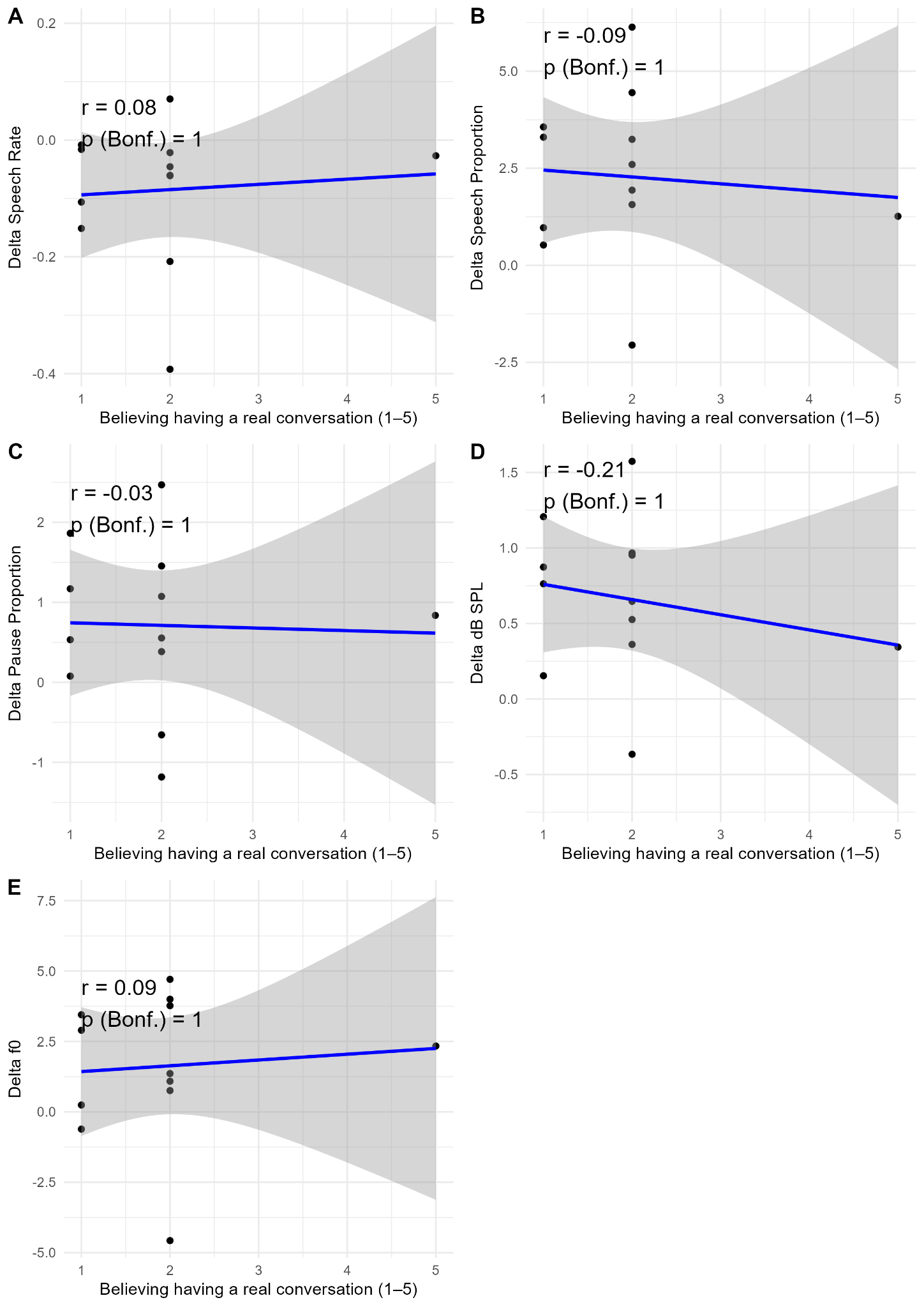
**

Supplementary Figure 2. Each panel shows the correlation between believing having a real conversation with a person seated in the adjacent room and the delta (i.e., change from the easy listening condition to the hard listening condition) in one of the five speech metrics: speech rate (A), speech proportion (B), pause proportion (C), vocal intensity (dB SPL, D), and fundamental frequency ($f_{0}$, E). Each plot includes Pearson's correlation coefficient (r) and the Bonferroni-corrected p-value. Regression lines with 95% confidence intervals are shown in blue.

**Impact of Cover Story Credibility on Speech Adaptations: Experiment 2**

**
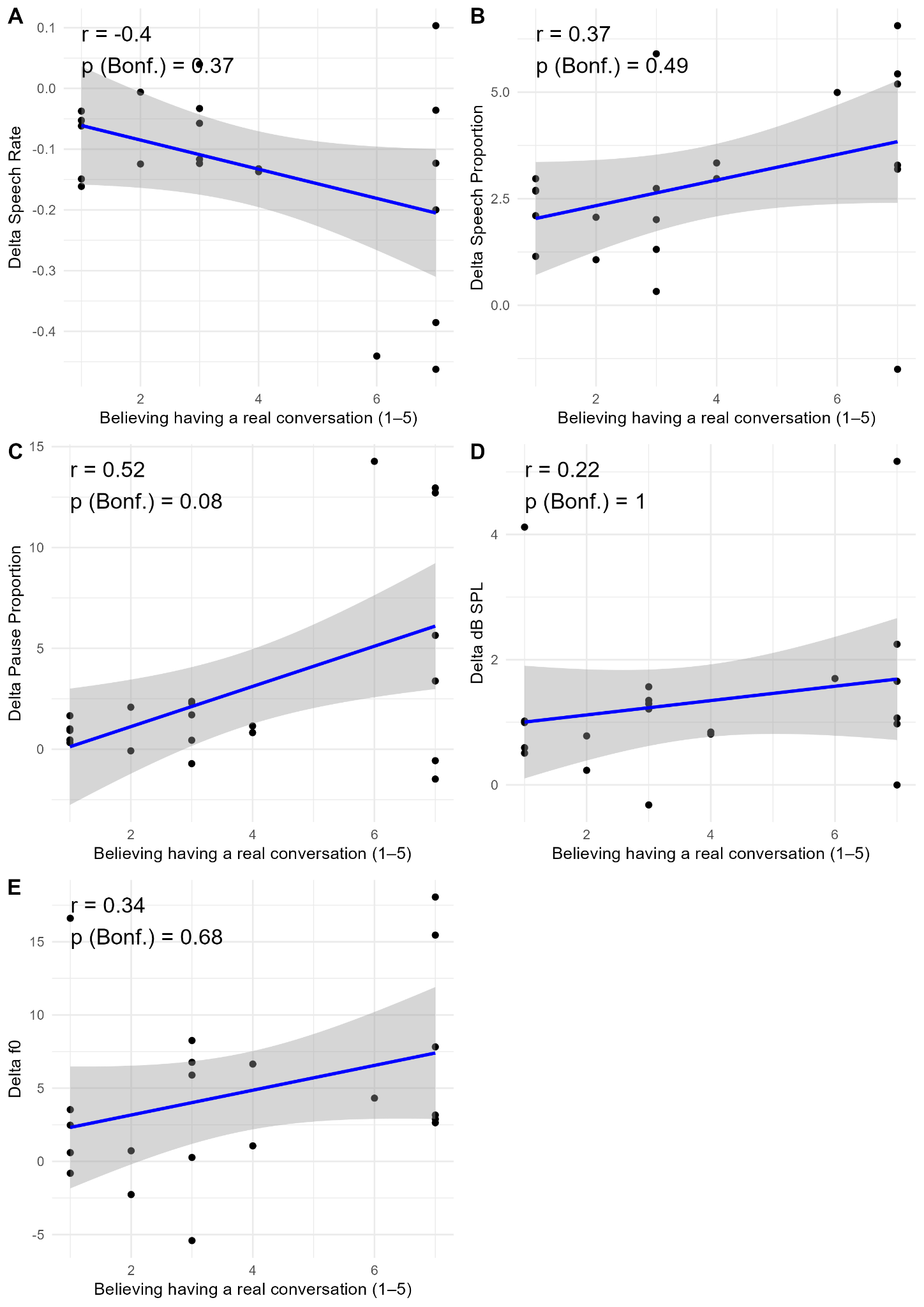
**

Supplementary Figure 3. Each panel shows the correlation between believing having a real conversation with a person seated in the adjacent room and the delta (i.e., change from the easy listening condition to the hard listening condition) in one of the five speech metrics: speech rate (A), speech proportion (B), pause proportion (C), vocal intensity (dB SPL, D), and fundamental frequency ($f_{0}$, E). Each plot includes Pearson's correlation coefficient (r) and the Bonferroni-corrected p-value. Regression lines with 95% confidence intervals are shown in blue.

**Combined analysis: comparing vocal adjustments across Experiment 1 and 2**

To address the non-significant trends in Experiment 2 and to increase the interpretability of the overall dataset, we performed combined Linear Mixed-Effects Models (LMEMs), executed in the statistical environment R via the lmer() function from the lme4 package. We assessed the combined effect of experimental manipulation by incorporating the factor experiment (1 vs. 2) into our LMEMs as a fixed effect, specifically testing the listening condition x experiment two-way interaction. To account for the within-subjects design, the model included random intercepts for participants, as well as random slopes for the listening condition by participant (i.e., a maximal model structure of listening condition | participant).

For the majority of dependent variables, neither a main effect of experiment nor the interaction between listening condition and experiment emerged (speech rate, main effect:$X^{2}$(1) = 1.74, *p* = .19, interaction: $X^{2}$(1) = 0.75, *p* = .39; speech proportion, main effect: $X^{2}$(1) = 1.73, *p* = .19, interaction: $X^{2}$(1) = 0.64, *p* = .42; pause proportion, main effect: $X^{2}$(1) = 1.59, *p* = .21, interaction: $X^{2}$(1) = 1.94, *p* = .16; voice intensity, main effect: $X^{2}$(1) = 0.83, *p* = .36, interaction: $X^{2}$(1) = 1.95, *p* = .09) The only exception was $f_{0}$ for which the main effect of experiment was significant ($X^{2}$(1) = 8.95, *p* = .003), indicating lower $f_{0}$ in Experiment 2 compared to Experiment 1. The interaction between listening condition and experiment was instead not significant ($X^{2}$(1) = 2.80, *p* = .09), suggesting that the change in $f_{0}$ was uniform across listening conditions.

**Categorical Re-Analyses of Listening Condition**

Experiment 1

Speech rate significantly decreased with an increase in visual cues of listening effort ($X^{2}$(1) = 16.54, *p* < .001). Similarly speech proportion ($X^{2}$(1) = 40.07, *p* < .001), pause proportion ($X^{2}$(1) = 15.70, *p* < .001), voice intensity ($X^{2}$(1) = 23.78, *p* < .001), and $f_{0}$ ($X^{2}$(1) = 12.94, *p* = .002) significantly increased as visual cues of listening effort increased.

When analysing whether acoustic parameters changed as a function of trial position within each mini-block, a non-significance pattern was found for speech rate (main effect: $X^{2}$(1) = 1.39, *p* = .24; interaction: $X^{2}$(1) = 1.98, *p* = .37), speech proportion (main effect:$X^{2}$(1) = 0.30, *p* = .58; interaction: $X^{2}$(1) = 1.29, *p* = .53), pause proportion (main effect:$X^{2}$(1) = 2.79, *p* = .10; interaction: $X^{2}$(1) = 1.46, *p* = .48), and for $f_{0}$ (main effect:$X^{2}$(1) = 0.29, *p* = .59; interaction: $X^{2}$(1) = 4.30, *p* = .12). For voice intensity, no main effect of trial position was found ($X^{2}$(1) = 0.07, *p* = .79), but the interaction with listening condition was significant ($X^{2}$(1) = 6.47, *p* = .04). Differences in vocal intensity across listening conditions became progressively more pronounced over successive trials, consistent with a gradual adaptation to listening demands. Specifically, the difference in intensity between the easy and hard listening conditions increased from approximately 1.0 dB at trial 1 to 1.7 dB at trial 5.

For what concerns the self-report measures, speech production changes were accompanied by increased speaking effort reported by participants as a function of the visible cues of listening effort ($X^{2}$(1) = 17.61, *p* < .001). Coherently with our manipulation, participants’ evaluations of the confederate’s listening effort also increased with visual cues of listening effort ($X^{2}$(1) = 38.06, *p* < .001; Figure 3B). Similarly, perceived speech comprehension of the confederate significantly decreased with an increase in visual cues of listening effort ($X^{2}$(1) = 49.72, *p* < .001).

Experiment 2

Consistent with the findings of Experiment 1, speech rate significantly decreased with an increase in visual cues of listening effort ($X^{2}$(1) = 6.28, *p* = .04). A similar pattern of increase with listening effort was found for speech proportion ($X^{2}$(1) = 16.77, *p* < .001) and voice intensity ($X^{2}$(1) = 18.13, *p* < .001). Conversely, the listening condition had no significant effect on pause proportion ($X^{2}$(1) = 3.13, *p* = .21) or $f_{0}$ ($X^{2}$(1) = 4.05, *p* = .13).

Regarding temporal adaptations, no significant main effect of trial position and no significant two-way interaction between trial position and the listening condition was observed for speech rate (main effect: $X^{2}$(1) = 0.50, *p* = .48; interaction: $X^{2}$(1) = 0.06, *p* = .97), speech proportion (main effect: $X^{2}$(1) = 0.24, *p* = .63; interaction: $X^{2}$(1) = 5.27, *p* = .07), pause proportion (main effect: $X^{2}$(1) = 1.27, *p* = .26; interaction: $X^{2}$(1) = 2.08, *p* = .35), and voice intensity (main effect: $X^{2}$(1) = 0.18, *p* = .67; interaction: $X^{2}$(1) = 0.37, *p* = .83). Conversely, a significant main effect of trial position was found for $f_{0}$ ($X^{2}$(1) = 4.44, *p* = .04), indicating a small but consistent change across trials within the mini-block. Inspection of model-predicted values revealed a slight decrease in $f_{0}$ from trial 1 (≈179.5 Hz) to trial 5 (≈178.3 Hz).

For self-report measures, speech production changes were accompanied by increased speaking effort reported by participants as a function of the visual cues of listening effort ($X^{2}$(1) = 6.67, *p* = .04). Coherently, participants’ ratings of the confederate’s listening effort also increased with visual cues of listening effort ($X^{2}$(1) = 69.40, *p* < .001). Similarly, perceived speech comprehension of the confederate significantly decreased with an increase in visual cues of listening effort ($X^{2}$(1) = 37.26, *p* < .001).

Combined analysis: comparing vocal adjustments across Experiment 1 and 2

For the majority of dependent variables, neither a main effect of experiment nor the interaction between listening condition and experiment emerged (speech rate, main effect:$X^{2}$(1) = 1.91, *p* = .17, interaction: $X^{2}$(1) = 1.73, *p* = .42; speech proportion, main effect: $X^{2}$(1) = 1.82, *p* = .18, interaction: $X^{2}$(1) = 1.14, *p* = .57; pause proportion, main effect: $X^{2}$(1) = 2.32, *p* = .13, interaction: $X^{2}$(1) = 1.99, *p* = .37; voice intensity, main effect: $X^{2}$(1) = 1.40, *p* = .24, interaction: $X^{2}$(1) = 2.95, *p* = .23) The only exception was $f_{0}$ for which the main effect of experiment was significant ($X^{2}$(1) = 9.44, *p* = .002), indicating lower $f_{0}$ in Experiment 2 compared to Experiment 1. The interaction between listening condition and experiment was instead not significant ($X^{2}$(1) = 5.40, *p* = .07), suggesting that the change in $f_{0}$ was uniform across listening conditions.
